# Identifying Triple-Negative Breast Cancer Using Background Parenchymal Enhancement Heterogeneity on Dynamic Contrast-Enhanced MRI: A Pilot Radiomics Study

**DOI:** 10.1371/journal.pone.0143308

**Published:** 2015-11-24

**Authors:** Jeff Wang, Fumi Kato, Noriko Oyama-Manabe, Ruijiang Li, Yi Cui, Khin Khin Tha, Hiroko Yamashita, Kohsuke Kudo, Hiroki Shirato

**Affiliations:** 1 Department of Radiation Medicine, Hokkaido University Graduate School of Medicine, North 15 West 7 Kita-ku, Sapporo, Hokkaido, 060–8638, Japan; 2 Department of Diagnostic and Interventional Radiology, Hokkaido University Hospital, North 14 West 5 Kita-ku, Sapporo, Hokkaido, 060–8648, Japan; 3 Department of Radiation Oncology, Stanford University School of Medicine, 291 Campus Drive, Li Ka Shing Building, Stanford, CA 94305, United States of America; 4 Global Station for Quantum Medical Science and Engineering, Global Institution for Collaborative Research and Education (GI-CoRE), Hokkaido University, Proton Beam Therapy Center, North 14 West 5 Kita-ku, Sapporo, Hokkaido, 060–8648, Japan; 5 Department of Breast Surgery, Hokkaido University Hospital, North 14 West 5 Kita-ku, Sapporo, Hokkaido, 060–8648, Japan; University of Chicago, UNITED STATES

## Abstract

**Objectives:**

To determine the added discriminative value of detailed quantitative characterization of background parenchymal enhancement in addition to the tumor itself on dynamic contrast-enhanced (DCE) MRI at 3.0 Tesla in identifying “triple-negative" breast cancers.

**Materials and Methods:**

In this Institutional Review Board-approved retrospective study, DCE-MRI of 84 women presenting 88 invasive carcinomas were evaluated by a radiologist and analyzed using quantitative computer-aided techniques. Each tumor and its surrounding parenchyma were segmented semi-automatically in 3-D. A total of 85 imaging features were extracted from the two regions, including morphologic, densitometric, and statistical texture measures of enhancement. A small subset of optimal features was selected using an efficient sequential forward floating search algorithm. To distinguish triple-negative cancers from other subtypes, we built predictive models based on support vector machines. Their classification performance was assessed with the area under receiver operating characteristic curve (AUC) using cross-validation.

**Results:**

Imaging features based on the tumor region achieved an AUC of 0.782 in differentiating triple-negative cancers from others, in line with the current state of the art. When background parenchymal enhancement features were included, the AUC increased significantly to 0.878 (*p*<0.01). Similar improvements were seen in nearly all subtype classification tasks undertaken. Notably, amongst the most discriminating features for predicting triple-negative cancers were textures of background parenchymal enhancement.

**Conclusions:**

Considering the tumor as well as its surrounding parenchyma on DCE-MRI for radiomic image phenotyping provides useful information for identifying triple-negative breast cancers. Heterogeneity of background parenchymal enhancement, characterized by quantitative texture features on DCE-MRI, adds value to such differentiation models as they are strongly associated with the triple-negative subtype. Prospective validation studies are warranted to confirm these findings and determine potential implications.

## Introduction

Breast cancer is a disease with several distinct biological subgroups [1,2]. Gene expression-based molecular subtyping is used clinically in the selection of the most appropriate therapy and has proved valuable for individualized management [3]. In particular, breast cancers that overexpress the estrogen receptor (ER), progesterone receptor (PR), and/or human epidermal growth factor 2 receptor (HER2) can be specifically targeted with hormonal and/or anti-HER2 therapies. Triple-negative (TN) breast cancers, however, lack expression of these three receptors, so currently have no targeted therapy available and are limited to general cytotoxic chemotherapies. TN cancers tend to be larger in size, are of higher grade, have lymph node involvement at diagnosis, and have the poorest prognosis [4–7]. The ability to differentiate TN cancers from other less aggressive subtypes using diagnostic imaging, could help identify and stratify patients with this rare and particularly difficult subtype for the appropriate therapy earlier than biopsy in the future.

Current methods of biopsy have limitations considering more than small samples of tissue, hence meet some issues with large and/or heterogeneous cancers [8]. MRI, however, provides anatomical and functional properties of whole tissues. Findings on MRI such as tumor size, morphology, shape, and enhancement characteristics (such as rim enhancement) have been shown as significant in differentiating breast cancer subtypes including TN breast cancers [9–12], though such manual annotation of tumor characteristics are generally limited to a few qualitative descriptors and are dependent on the operator [13]. On the other hand, computer-aided diagnosis (CAD) has paved the way to improve diagnostic specificity by computing quantitative information about the entire tumor non-invasively in an objective manner and reducing inter-reader variability [14–17]. More recently, the radiomics approach of CAD has emerged with the central premise that cancer imaging phenotypes reflect underlying gene expression patterns and combining these sources of information will improve individualized treatment selection and monitoring [18–20]. The approach has shown great promise considering whole tissues relatively comprehensively by automatically extracting and evaluating large sets of advanced quantitative imaging features, including texture heterogeneity patterns [21].

To date, most breast cancer studies have focused on characterization of the tumor itself [13–17,22–26]. Relatively little is known about the diagnostic and prognostic significance of its surrounding parenchyma tissue on MRI. Initial studies indicate that increased background parenchymal enhancement (BPE) on dynamic contrast-enhanced MRI (DCE-MRI) could lead to higher rates of misinterpreting benign tissues as suspicious [27,28], though the precise reasons for this enhancement are not clear. There is also evidence suggesting that tumor microenvironment may help define and regulate breast cancer progression [29–32], as well as predict disease recurrence following therapy [33]. Additionally, the appearance of BPE on MRI and parenchyma on mammography have been associated with risk of developing breast cancer independently [34–39]. We hypothesize BPE may also have prognostic significance with breast cancer subtype.

The purpose of this study was to determine the added discriminative value of detailed quantitative characterization of BPE in addition to the tumor itself on DCE-MRI at 3.0 Tesla in identifying TN breast cancers. Our work was based on semi-automated, volumetric segmentation of the tumor and its surrounding parenchyma. We extracted a variety of quantitative imaging features of both regions in 3-D on DCE-MRI in addition to standard radiologist-evaluated clinical features and combined them with machine learning tools to obtain the optimal subtype classification.

## Materials and Methods

### Study Population

Eighty-four women, presenting 88 lesions pathologically proven as invasive carcinoma, were enrolled in the study. All underwent DCE-MRI before their surgical procedure in the period of February 2012 to May 2013 and had pathology reports with molecular subtype results available. Four women with multiple lesions (one in each breast, treated as separate cases) were included. Those found with multiple unilateral lesions, however, were excluded. Additional criteria for exclusion from the study included having received neoadjuvant chemotherapy, hormonal therapy, or having artifacts on MRI exams. Patient demographics are summarized in Table 1. This retrospective study was approved by the Institutional Review Board of Hokkaido University Hospital and informed consent was waived according to Ethical Guidelines for Clinical Studies of the Japanese Ministry of Health, Labour, and Welfare. All patient data were anonymized and de-identified prior to analysis.

**Table 1:** Patient and subtype demographics.

Parameter	All	By presence of receptor			By St. Gallen consensus [3]		
		ER+	PR+	HER2+	TN	LumA	LumB
**Patients, n**	84	69	60	4	11	42	27
**Lesions, n**	88	73	63	4	11	45	28
**Mean age, years**	59.1 (11.0)	58.8 (10.7)	58.9 (11.0)	60.0 (10.2)	61.2 (12.3)	59.1 (9.9)	58.4 (12.1)
**Mean BMI**	23.2 (4.1)	23.5 (4.3)	23.9 (4.3)	22.5 (1.5)	22.3 (4.9)	23.3 (4.5)	23.9 (4.0)
**Postmenopausal patients, n**	61	51	42	4	10	33	18

Some overlap exists between subtypes defined and tumors may be represented in more than one category. Classification criteria are described in the Pathological Subtyping section of the methods. Standard deviations are displayed in parentheses with mean measures. BMI = body mass index, TN = Triple-negative, LumA = Luminal A, LumB = Luminal B.

### Pathological Subtyping

Expression of ER, PR, HER2 and Ki67, a marker of cellular proliferation and subtype [40,41], were determined by immunohistochemical analysis of tumor specimens. Each tumor sample was classified as ER+, PR+, and/or HER2+, or being triple-negative (TN) if negative for all three. Additionally, cancers were classified as Luminal A (LumA, if ER+ and/or PR+, HER2-, Ki67<14%) or Luminal B (LumB, if ER+ and/or PR+, HER2 over-expressed or Ki67≧14%), as also clinically relevant in individualized therapy [3]. Tumor subtype demographics are also summarized in Table 1.

### Image Acquisition

MR imaging was performed using one Achieva 3.0T TX system (Philips Healthcare, Best, Netherlands) with a 7-channel breast coil while patients lied prone. The dynamic protocol used was in accordance with European Society of Breast Imaging [42] and American College of Radiology guidelines [43]. In brief, 3-D T1-weighted images were acquired bilaterally in the axial plane with a fat-suppressed gradient echo sequence (e-Thrive): Repetition time/echo time 4.9 ms/2.4 ms, flip angle 10°, field of view 320 × 320 mm, voxel size 0.8 × 0.8 × 1.6 mm (reconstructed 0.8 mm isovoxel), and SENSE parallel imaging factor 2.4. Images at four time points were acquired, each lasting one minute. The first image was taken immediately before injection of contrast material (Gadopentetic acid with diethylenetriaminepentacetate, 0.1 mmol/kg) and flushing with 20mL saline (t1), the second and third in the early phase at 1 and 2 minutes after injection (t2 and t3 respectively), and the last in the late phase at 6 minutes after injection (t4).

### Image Segmentation and Feature Extraction

MR images were reviewed retrospectively by a board-certified radiologist specializing in breast MRI with 13 years of experience (F.K.), blinded of findings other than diagnosis as invasive breast cancer. Clinical features concerning tumor morphology were evaluated according to Breast Imaging Reporting and Data System (BI-RADS) MRI [44]. For mass lesions: shape, margin, and internal enhancement characteristics were evaluated; and for non-mass lesions: distribution and internal enhancement characteristics were evaluated. Morphology and mass size (mass lesions via longest axis) were also included in the analysis.

**Fig 1:**
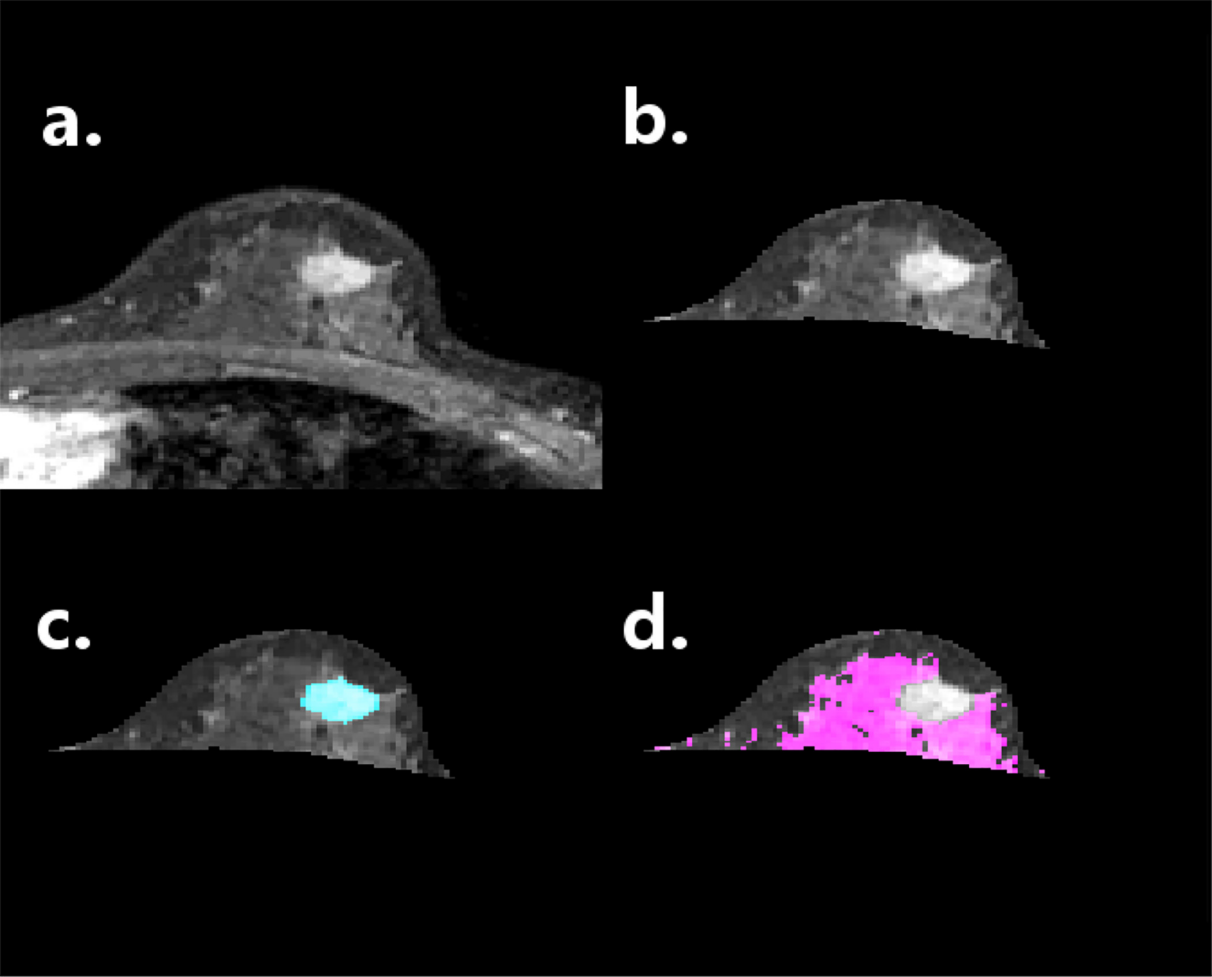
Example of tissue segmentation performed of all cancer patients’ affected breast images. At top left (a), a dynamic contrast-enhanced MRI exam at t3 is seen in the axial plane, illustrating one slice of the view used for contouring the breast and tumor. At top right (b), the result of breast segmentation is shown. At bottom left (c), the segmented tumor is highlighted in blue. Finally at bottom right (d), the parenchyma segmented at t1 is highlighted in pink. Breast subcompartment segmentation was performed in 3-dimensions.

Contouring of the affected breast was performed using images acquired at t3 (near max intensity for all tissues) with automatic detection of the skin edge by thresholding plus semi-automatic delineation of the chest wall and nipple at every slice by interactive placement of an expanding polygon mask. The breast tumor was also segmented at t3, to better distinguish it from background parenchyma, using a semi-automated gray-level intensity threshold 3-D region-growing technique [45] that was manually modified as necessary. Subsequently, separation of the remaining non-tumor breast tissue into fibroglandular parenchyma and adipose tissue compartments was performed semi-automatically at t1 (pre-contrast, as recommended for assessment of fibroglandular tissue in latest edition of BI-RADS atlas) [44], using an adapted fuzzy *c*-means clustering technique [46]. The unsupervised algorithm assigns a membership to each voxel initiated with user-seeding and automatically determines a threshold best to separate parenchyma and adipose tissue. An illustration of tissue compartment segmentation is shown in Fig 1. At this point, breast density was calculated as the percentage of breast volume that was made up of parenchyma and included in the analysis. All subsequent features were extracted of the tumor and parenchyma compartments.

Three standard pharmacokinetic parametric maps were generated from each DCE-MRI to capture enhancement quantitatively: contrast material rate in (from t1 to maximum), percent enhancement (PE, from t1 to maximum), and signal enhancement ratio (SER, change from t1 to maximum relative to change from t1 to t4) [47].

Four first-order statistical features were calculated from the parameter maps of the tumor and parenchyma compartments: mean, standard deviation, skewness, and kurtosis. Nine second-order statistical features, also known as gray-level co-occurrence texture features [48], were calculated at a 1-voxel distance offset of the maps and averaged across the 26-directions of 3-D space after rescaling to 8-gray level (3-bit) data: energy, contrast, correlation, variance, homogeneity, sum mean, entropy, inertia, and cluster shade. Thirteen statistical textures of 3 parametric maps resulted in 39 features capturing enhancement heterogeneity for each tissue compartment studied, which were included in the analysis. Those of the parenchyma are defined here also as BPE texture or heterogeneity. All image processing was performed using MATLAB R2012b (Mathworks, Inc., Natick, MA, USA) software.

### Predictive Modeling for Differentiation

Based on the extracted imaging features, we aimed to distinguish TN breast cancers from other subtypes using machine learning tools. In particular, we performed 5 classification tasks: differentiating TN cancers against non-TN, ER+, PR+, LumA, and LumB cancers. Differentiating TN from HER2+ cancers was not performed as the number of tumors combined from these groups was insufficient given techniques used (n = 15).

To minimize bias in our evaluation, stratified 10-fold cross-validation was performed [49]. 10 bootstrap repetitions of each were run, from which performance metrics (described below) were averaged and confidence intervals were estimated. A two-step feature selection technique was applied on imaging features before classification. First, features were ranked by the *X* ^2^ statistic [50] to identify strength of association with the subtype in question; second, a sequential forward floating search algorithm was used to identify a small subset of optimal features large enough to capture data complexity [51]. Finally, a support vector machine (SVM) classifier was trained [52,53] from the selected feature subset of preceding search steps. SVM models are non-probabilistic binary linear classifiers, which represent the data in higher dimensionality spaces, mapped so as to separate the categories with a divide that is as wide as possible. Both steps of feature selection were encapsulated with the classifier within each training fold in order to avoid feature selection bias and overfitting [53,54]. Feature selection and classification were also performed with regularization, which served to penalize model complexity, as another measure to avoid overfitting [53]. All predictive modeling was performed using Waikato Environment for Knowledge Analysis (WEKA) 3.6.12 (University of Waikato, Hamilton, New Zealand) [55].

### Cluster Analysis

In addition to the supervised approach (differentiating subtype-labeled data) in the classification modeling described above, an unsupervised approach was also taken with learning tasks. Clustering of BPE texture features in a *k*-means manner, as used with gene analysis to reveal groups with similar expression patterns [56,57], was performed without using knowledge of subtype. All BPE texture features of included cases were normalized as *z*-scores and clustered into two partitions using genomic data analysis framework Gitools 2.2.1 (Universitat Pompeu Fabra, Barcelona, Spain) [58].

### Statistical Analysis

**Fig 2:**
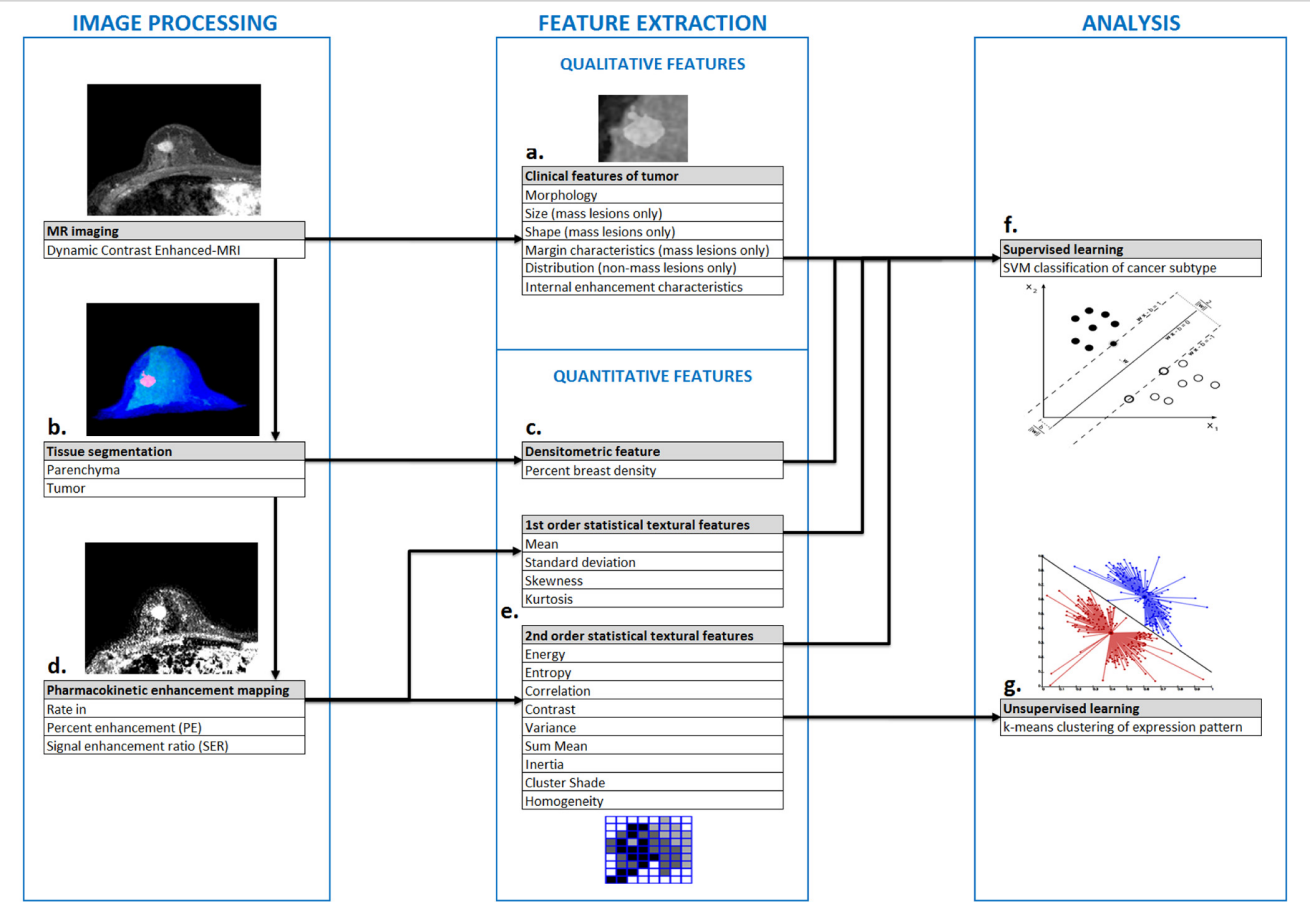
Summary of radiomic analysis performed in this study. Clinical features were evaluated by a radiologist according to Breast Imaging Reporting and Data System directly from dynamic contrast-enhanced MRI (a). 3-Dimensional tumor (red) and parenchyma (light blue) compartments were segmented (b), from which volumetric breast density was immediately estimated (c). Enhancement maps were then generated (d), from which textural features of tissue compartments were extracted and defined as enhancement heterogeneity (e). Subsequently, two analyses were conducted using extracted features: supervised learning of breast cancer subtype was performed with a support vector machine classifier (f) and unsupervised learning of background parenchymal enhancement feature expression pattern was performed with *k*-means clustering (g).

Fig 2 summarizes the study’s radiomic analysis performed as described above. Classification performance of predictive modeling was evaluated using accuracy, sensitivity, specificity, and area under the receiver operating characteristic curve (AUC) values averaged over all bootstrap folds of cross-validation. Wilcoxon signed-rank tests were used to test significance of paired difference between classification models’ performance without inclusion of BPE-derived features against those with in a non-parametric manner. *p*-values of less than 0.05 were interpreted as significant. All statistical analyses were performed using JMP 11.0.0 (SAS Institute Inc., Cary, NC, USA).

## Results

### Differentiation of Molecular Subtypes

Performance metrics for subtype differentiation tasks are detailed in Table 2. Classification models using both tumor and parenchyma features generally outperformed those based only on tumor features, most notably in terms of accuracy, sensitivity, and AUC. The most remarkable discriminative performance was seen in the classification of TN against ER+ cancers. While the conventional model based on tumor features achieved an AUC of 0.780, the model using both parenchyma and tumor features improved AUC significantly to 0.883 (p<0.01). Accuracy and sensitivity improved from 86.3% to 89.4% (p<0.01) and 35.5% to 62.0% (p<0.01) respectively with this task. Performance classifying TN against all other cancers improved similarly from an AUC of 0.782 to 0.878 (p<0.01) by including BPE texture features, with accuracy and sensitivity improved from 86.9% to 90.0% (p<0.01) and 33.0% to 57.0% (p<0.01) respectively. Classifying TN against PR+ cancers improved from an AUC of 0.731 to 0.859 (p<0.01), with accuracy and sensitivity improved from 83.5% to 87.8% (p<0.01) and 28.5% to 53.0% (p<0.01) respectively.

**Table 2:** Performance results of predictive modeling.

Differentiation task	n	Using tumor features				Using both tumor & BPE features			
		Accuracy, %	Sensitivity, %	Specificity, %	AUC	Accuracy, %	Sensitivity, %	Specificity, %	AUC
**TN vs others**	**88**	86.9 (85.1, 88.7)	33.0 (23.8, 42.2)	94.7 (92.9, 96.4)	0.782 (0.730, 0.833)	90.0* (88.1, 91.8)	57.0* (47.4, 66.6)	94.7 (93.0, 96.2)	0.878* (0.838, 0.918)
**TN vs ER+**	**84**	86.3 (84.2, 88.3)	35.5 (26.1, 44.9)	94.1 (92.3, 95.8)	0.780 (0.730, 0.830)	89.4* (87.5, 91.3)	62.0* (52.7, 71.3)	93.6 (91.7, 95.4)	0.883* (0.843, 0.923)
**TN vs PR+**	**74**	83.5 (81.4, 85.6)	28.5 (19.8, 37.2)	93.0 (90.7, 95.3)	0.731 (0.674, 0.788)	87.8* (85.7, 90.0)	53.0* (43.2, 62.8)	94.1 (92.2, 95.9)	0.859* (0.817, 0.901)
**TN vs LumA**	**56**	79.6 (76.9, 82.3)	40.5 (30.9, 50.1)	88.8 (85.8, 91.8)	0.795 (0.745, 0.844)	81.8 (78.6, 85.1)	49.5 (39.8, 59.2)	89.8 (86.8, 92.8)	0.814 (0.756, 0.872)
**TN vs LumB**	**39**	61.3 (57.5, 65.2)	29.0 (20.3, 37.7)	73.8 (68.5, 79.2)	0.635 (0.577, 0.693)	84.3* (80.7, 87.8)	69.5* (60.7, 78.3)	90.0* (86.2, 93.8)	0.789* (0.728, 0.850)

Metrics displayed as: mean (95% confidence interval). TN = Triple-negative, ER = estrogen receptor, PR = progesterone receptor, HER2 = human epidermal growth factor 2 receptor, LumA = Luminal A, LumB = Luminal B, BPE = background parenchymal enhancement, AUC = area under receiver operating characteristic curve.

*p<0.01 by Wilcoxon signed-rank test in comparing models including use of both tumor and BPE features against those using only tumor features.

In differentiating TN against LumB cancers, a significant improvement by including BPE texture features in terms of specificity from 73.8% to 90.0% (p<0.01) was also apparent in addition to improvements in accuracy, sensitivity, and AUC from 61.3% to 84.3% (p<0.01), 29.0% to 69.5% (p<0.01), and 0.635 to 0.789 (p<0.01) respectively. Including parenchyma features did not significantly improve the ability to differentiate TN against LumA cancers, where performance using only tumor features already achieved an AUC of 0.795.

### Optimal Imaging Features

The most discriminating features selected in each classification task’s cross-validation process are summarized in Table 3, indicated with percentage of cross-validation folds in which they were selected as a simple indicator of significance. The Supplemental Table (S1 Table) elaborates on this further by detailing distributions of selected feature values as well as their SVM weights, whose magnitude indicates prognostic value. In tumor feature-based models, standard clinical features such as mass size and mass shape proved to be most prevalent in differentiating TN from all other subtype groups, having been selected in all tasks as the top features. Mass margin characteristics also proved to be prevalent in several models, as did internal enhancement characteristics. Tumor morphology and breast density were discriminative in a couple of models each. Tumor enhancement texture features also proved to be prevalent across many differentiation tasks performed. Specifically, ‘mean of tumor SER’ and ‘inertia of tumor rate in’ proved to be effective discriminators of TN against all other, ER+, and PR+ subtypes. Several other enhancement texture features, derived from tumor PE, proved discriminative across each tumor-based models performed. Enhancement textures derived from tumor rate in were also selected classifying all other and ER+ cancers from the TN subtype.

**Table 3:** Optimal imaging features selected. Imaging features most discriminative in prediction models, using tumor features (top 5 rows) or using both tumor and parenchyma features (bottom 5 rows). Features that survived the selection algorithm in the majority of cross-validation folds of the given task are shown with numbers indicating percentage of folds in which the feature was selected as a simplistic indicator of significance (see Supplemental Table, S1 Table, for further elaboration of feature values and their significance). Features are individually identified here at the bottom of the table, abbreviated by type and defined in the footnote.

Model type	Differentiation task	Feature type																						
		Tumor					Breast	Tumor enhancement										BPE						
		Clinical					Densitometric	1st order texture				2nd order texture						1st order texture		2nd order texture				
** **	**TN vs others**		100%	99%	89%	85%			64%		95%	59%		76%										
** **	**TN vs ER+**		100%	99%	86%	82%	75%			55%	93%		62%	70%			61%							
**tumor**	**TN vs PR+**	62%	100%	97%			91%	64%			88%													
** **	**TN vs LumA**	52%	100%	93%		96%										75%								
** **	**TN vs LumB**		79%	79%	51%										54%									
** **	**TN vs others**			98%														100%	100%		69%		69%	67%
** **	**TN vs ER+**			96%														100%	100%		62%		62%	58%
**tumor + BPE**	**TN vs PR+**			92%				72%										96%	100%			66%		
** **	**TN vs LumA**			79%														89%	98%					
** **	**TN vs LumB**														87%				100%	74%				95%
** **	** **	T1	T2	T3	T4	T5	B1	TE1	TE2	TE3	TE4	TE5	TE6	TE7	TE8	TE9	TE10	PE1	PE2	PE3	PE4	PE5	PE6	PE7
** **	** **	**Features**																						

TN = Triple-negative, LumA = Luminal A, LumB = Luminal B, BPE = background parenchymal enhancement, PE = percent enhancement, SER = signal enhancement ratio.

T1 = morphology, T2 = mass size, T3 = mass shape, T4 = mass margin characteristics, T5 = internal enhancement characteristics. B1 = breast density. TE1 = tumor PE mean, TE2 = tumor PE std, TE3 = tumor PE skewness, TE4 = tumor SER mean, TE5 = tumor rate in Energy, TE6 = tumor rate in Homogeneity, TE7 = tumor rate in Inertia, TE8 = tumor PE Variance, TE9 = tumor PE Homogeneity, TE10 = tumor PE Inertia. PE1 = parenchyma rate in std, PE2 = parenchyma SER skewness, PE3 = parenchyma rate in Energy, PE4 = parenchyma PE Homogeneity, PE5 = parenchyma PE Entropy, PE6 = parenchyma PE ClusterShade, PE7 = parenchyma SER Variance.

When parenchyma features were used in addition to those of the tumor in modeling cancer subtype, BPE texture features overshadowed standard clinical, breast density, and tumor enhancement features almost completely as most prevalent in nearly all differentiation tasks performed. As seen in Table 3, though mass shape remained an effective discriminator of TN against all other, ER+, and PR+ cancers, it appears BPE features ‘skewness of parenchyma SER’ and ‘standard deviation of parenchyma rate in’ were by far the most prevalent predictors in all differentiation tasks. ‘Variance of parenchyma SER’, ‘homogeneity of parenchyma PE’, and ‘cluster shade of parenchyma PE’ proved to be effective discriminators of TN vs all other, ER+, and LumB cancers. Two texture features of tumor enhancement, ‘mean’ and ‘variance of tumor PE’, remained in models differentiating TN against PR+ and LumB cancers respectively, as were previously selected in tumor-based models differentiating the same.

**Fig 3:**
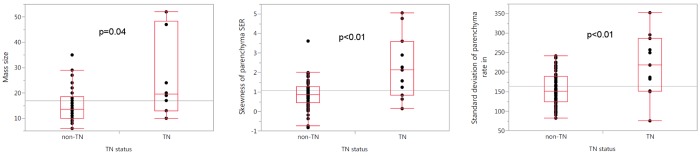
Box plots illustrating differences in distributions (quartiles as red boxes, grand mean indicated as spanning line) of the three most predictive quantitative features found in differentiation tasks: the lesion’s ‘mass size’ feature (a), parenchyma’s ‘skewness of Signal Enhancement Ratio’ feature (b), and parenchyma’s ‘standard deviation of rate in’ feature (c) compared between the triple-negative (TN) and non-TN groups. p-values were calculated by Wilcoxon Mann-Whitney tests.

**Fig 4:**
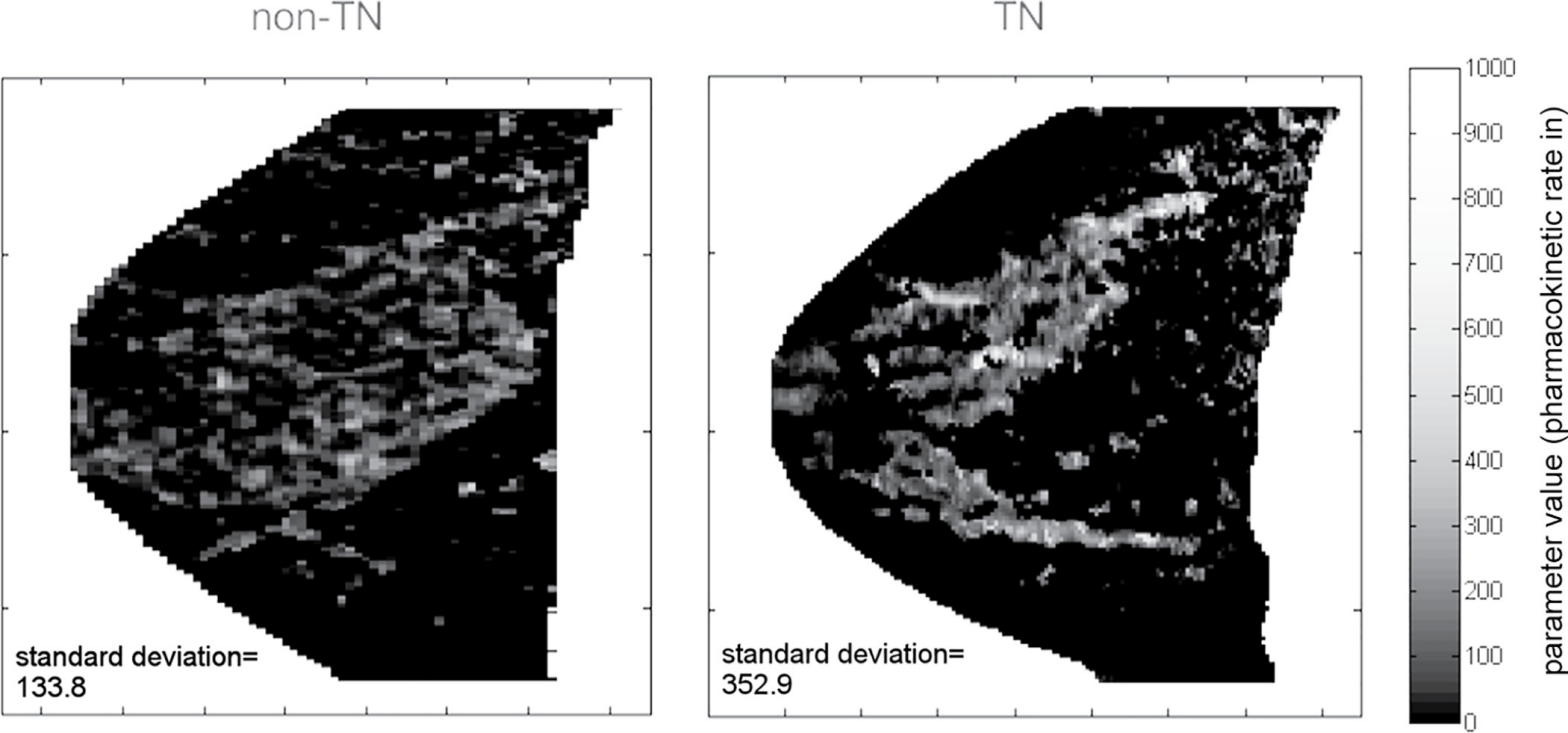
Examples of ‘parenchyma rate in’ parameter (also Fig 3B) maps from a non-triple-negative (TN) patient (left) and a TN patient (right) illustrating the difference of a statistical texture feature between members of the two groups in image form. Slices of the ‘parenchyma rate in’ parameter map void of tumor tissue are presented in the sagittal plane. It is evident the variation of this background parenchymal enhancement texture feature’s value is greater in TN cancers, where standard deviation is markedly higher at 352.9 as opposed to 133.8 in the non-TN patient.

Three of the most discriminative tumor and parenchyma features selected in comparisons between TN and non-TN cases are presented in Fig 3. Box plots illustrate the distributions of the three most predictive quantitative features found in differentiation tasks, and the differences in these distributions between the TN and non-TN group. Fig 4 presents visualizations of the BPE feature ‘standard deviation of parenchyma rate in’ (also Fig 3B), in the form of pharmacokinetic enhancement maps, illustrating the difference of this BPE texture between examples of one non-TN (left) and one TN (right) patient.

### Clustering

**Fig 5:**
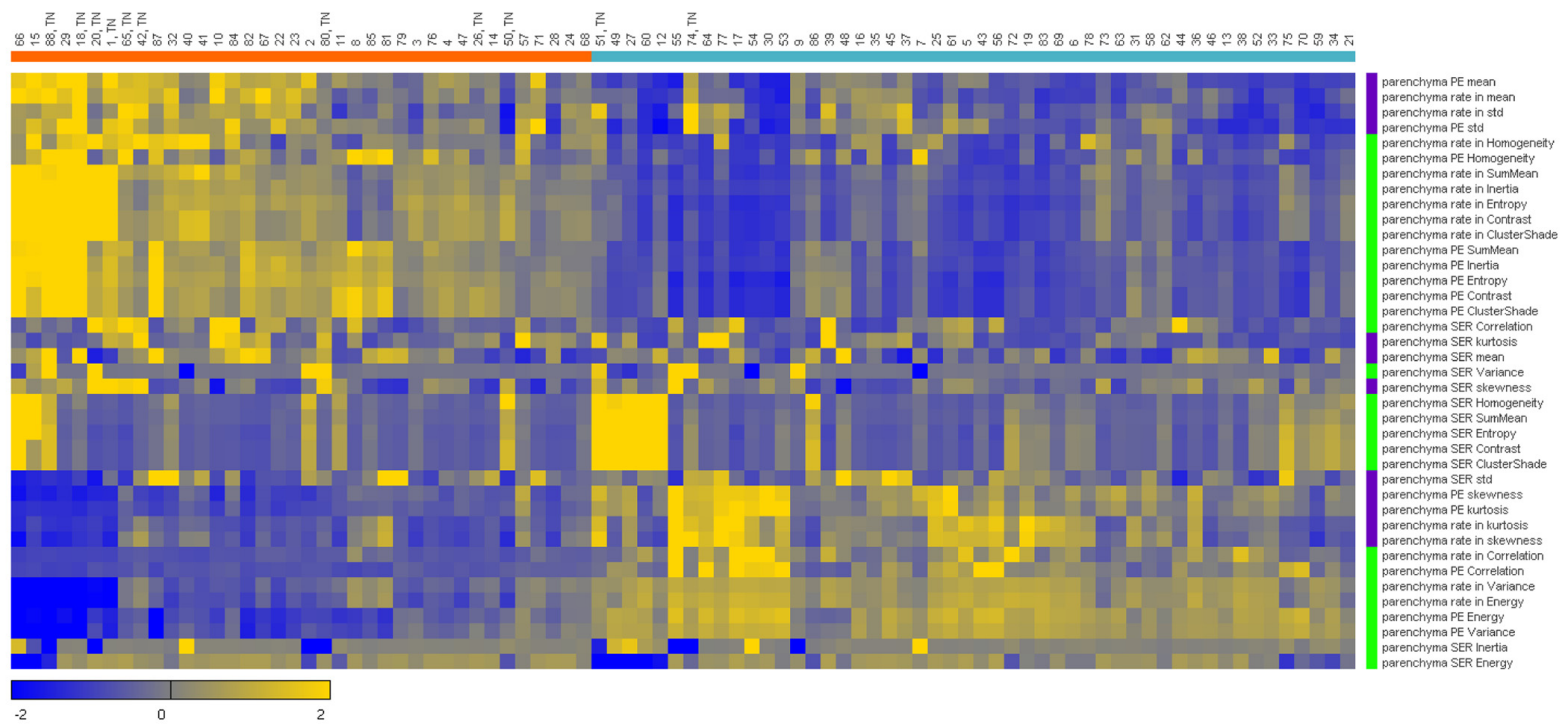
Unsupervised *k*-means clustering of breast cancer patients (n = 88) on the x-axis and quantitative background parenchymal enhancement (BPE) feature expression (n = 39) on y-axis (as z-scores, with scale at bottom left. std = standard deviation). Correspondence of patient groups with similar radiomic expression patterns can be seen where the majority of triple-negative (TN) breast cancers have grouped together in the left cluster (9 of 11 TN in partition highlighted orange at top left) due to association of the BPE heterogeneity feature signatures. 1^st^ order statistical texture features are highlighted as purple and similarly 2^nd^ order statistical texture features are green at right indicating correspondence of feature groups with clustered expression patterns.

A graphical heatmap representation of clustering results can be seen in Fig 5. Unsupervised *k*-means clustering of cases into two partitions (highlighted orange and turquoise) based on BPE texture features resulted in TN breast cancers showing a much higher presence in one partition than the other, with 9 of 11 cases clustering together (left, highlighted orange bar). Values are represented as *z*-scores illustrating the distributions of each feature and the signatures of features across cases.

## Discussion

Our study demonstrates that differentiation of breast cancer subtype with DCE-MRI can be improved by CAD systems exploiting features of the surrounding parenchyma tissue. In predictive classification models based on imaging features of the tumor, we see performance metrics on the order with the current state of the art in the 0.7–0.8 range [25,26]. Based on our results, adding quantitative imaging features of BPE greatly improves the discriminative ability of such prediction models, bringing performance up to 90.0% accuracy and AUC up to 0.883. Unsupervised clustering of BPE texture features into two partitions also revealed a significant association between BPE heterogeneity and TN status. 9 of 11 TN cancers in our study grouped together in one partition based only on BPE features, reinforcing the notion that BPE heterogeneity on DCE-MRI has a strong relationship with TN breast cancers

To take the functional portion of breast tissue into consideration with the radiomics approach of extracting comprehensive amounts of imaging features appears to be an improvement over conventional methods of tumor-based image phenotyping. While the standard tool to prove subtype in breast cancer remains to be tissue biopsy, we add to the growing understanding of imaging’s clinical importance and expand the role of MRI in more personal approaches of breast cancer diagnosis. Beyond considering information from a limited portion of the tumor with its inherent issues with false negatives especially when it comes to large or heterogeneous targets [8], imaging has the ability to provide information on entire tissues and their heterogeneity in a non-invasive manner. This ability appears valuable clinically in differentiating molecular subtypes of breast cancer. To our knowledge, this is the first study to demonstrate that quantitative texture features of BPE extracted from routine MRI are strongly predictive of TN breast cancers.

Our findings appear consistent with recent developments toward the tumor’s local environment being gradually recognized as a key contributor in breast cancer progression and aggressiveness [29,30]. Likewise, Pathak et al. [59] demonstrated that *in vivo* MRI specifically, could non-invasively monitor changes in tumor microenvironment, which could predict the cancer’s ability to metastasize.

Our findings also appear consistent with recent works showing an association between BPE and breast cancer diagnoses [34,39]. Existing evidence has linked increased BPE levels with greater hormonal activity, particularly estrogen [60–62], and these two studies suggest BPE could be a stronger predictor of breast cancer risk and potentially serve as an imaging biomarker of estrogen responsive malignant transformation. King et al. allude to presence of cancer having some systemic effects causing increased BPE and Dontchos et al. elaborate on this further, acknowledging the possibility BPE is a marker of physiologically active tissue more prone to tumorigenesis. Besides being consistent with associations found in our study, such concepts appear to be supported by work linking local inflammation and breast cancer transformation [63]. These findings, in support of our own, suggest that BPE could potentially help physicians better tailor screening and management strategies with breast cancer. This is important as we move into an era of more personalized approaches to medicine.

Furthermore, in a 2014 radiogenomic study, Mazurowski et al. reported on the relationship between MRI enhancement dynamics of the tumor and parenchyma to LumB cancer [64]. Though we did not include the same measure explicitly in our analysis, our findings reflect tumor and parenchyma enhancement characteristics both play significant roles in differentiating LumB cancers, in our case against TN cancer.

Our findings appear to be inconsistent with those of Ahn et al. [65], who concluded no association between BPE and aggressiveness of the primary cancer. Though in their study, the method of BPE quantification differed and only postmenopausal woman were enrolled, our findings indicate BPE is predictive of the more aggressive TN cancers against other subtypes. We attribute our discovery to having been able to capture functional activity of a tumor’s active microenvironment related to tumor progression [29–32] more broadly via extensive measures of enhancement texture.

It is perhaps interesting to note the presence of ‘mean of tumor SER’ as important in differentiating all other, ER+, and PR+ cancers against TN in tumor feature-based models. In 2011, Arasu et al. showed use of SER volume parameters on MRI were significantly associated with malignancy and improved diagnostic specificity without affecting sensitivity [47]. The following year Hylton et al. showed tumor SER as a stronger predictor of pathologic response to neoadjuvant chemotherapy than clinical assessment [66]. We elaborate on the utility of volumetric tumor SER in predicting molecular subtype of breast cancer.

It is also perhaps interesting to discuss the presence of breast density as important in differentiating ER+ and PR+ cancers against TN in tumor feature-based models. As it has been shown to be largely in agreement with mammographically-derived density measures [67], MRI-derived density also appears to be associated with breast cancer subtypes and tumor aggressiveness [68]. Though the predictive value of this specific feature in our study appears to be outweighed by that of BPE texture when available (breast density was no longer selected in models which also made use of parenchyma enhancement features in our study), it is apparent quantitative measures relating to parenchyma tissue may have prognostic value with breast cancer subtype on DCE-MRI. Bearing in mind that breast density is a limiting factor for cancer detection using mammography [36,69] and dense-breasted women are currently recommended to be stratified for supplemental screening with MRI to compensate for this [70,71] further emphasizes the potential value of considering the parenchyma in diagnostic imaging and predictive modeling of breast cancer.

Compared with another recent study on identifying TN cancers using quantitative image analysis by Agner et al. [26], our work has several strengths. First, we investigated not only tumor features but also BPE features for predicting TN breast cancers. Second, our imaging features were obtained by 3-D semi-automated segmentation of the tumor while Agner’s approach was based on 2-D manual segmentation. Semi-automated image analysis can reduce inter-observer variations and be scaled up relatively easily [72]. Both of which are critical components of the radiomics approach. Finally, using the increased signal-to-noise ratio of 3.0T MRI exclusively may have also contributed to the improved classification performance of our study.

Our study has several limitations as well. First, it is a relatively small, retrospective study. Larger prospective validation studies are warranted to confirm these findings and determine potential implications. Also, our method for semi-automated tumor segmentation was at times imperfect. Expert review of each MRI slice and appropriate correction was performed to best separate tumor from the background parenchyma, but the technique leaves some room for improvement. Lastly, due to the relatively large number of image features and exponentially increasing computing constraints involved, it was not within the scope of this study to perform exhaustive searches for optimal combinations of features. Here we adopted an efficient, locally optimal selection technique (forward floating search), that takes interaction between variables into account.

In conclusion, we demonstrated that quantitative image phenotyping of breast tumors and their surrounding parenchyma on DCE-MRI could distinguish TN breast cancers from other subtypes with higher accuracy than considering characteristics of the tumor alone. This is due to heterogeneity of background parenchymal enhancement characterized by texture on DCE-MRI being strongly associated with TN cancers. Considering heterogeneity of the tissue surrounding cancer in addition to the cancer itself could make for more sensitive and comprehensive differentiation of breast cancer subtype.

## Supporting Information

S1 Dataset Data underlying findings reported in study. All data underlying the findings reported in our study for public access. Rows list lesions included in the study and columns list (from left to right) clinical features read by the radiologist, tumor enhancement texture features, background parenchymal enhancement texture features, and subtype classifications.
(CSV)

S1 Table Optimal imaging feature distribution and prognostic significance. Details of imaging features selected as most discriminative in prediction models. Feature values (or numbers, with categorical features) are presented with their support vector machine (SVM) weights. Bootstrap estimates of SVM weights indicate prognostic significance, where the larger in absolute magnitude the weight, the more significant the feature (features were normalized with use of SVM classifier, though are presented here in their original scale). Features are listed alphabetically by differentiation task and model type. TN = Triple-negative, LumA = Luminal A, LumB = Luminal B, SVM = support vector machine, BPE = background parenchymal enhancement, std = standard deviation, n/a = not applicable, PE = percent enhancement, SER = signal enhancement ratio.
(XLSX)
